# Protective role of *Chlorella vulgaris* with Thiamine against Paracetamol induced toxic effects on haematological, biochemical, oxidative stress parameters and histopathological changes in Wistar rats

**DOI:** 10.1038/s41598-021-83316-8

**Published:** 2021-02-16

**Authors:** Amera Abd El Latif, Doaa H. Assar, Ebtihal M. Elkaw, Hanafy A. Hamza, Dalal Hussien M. Alkhalifah, Wael N. Hozzein, Ragaa A. Hamouda

**Affiliations:** 1 Department of Pharmacology, Faculty of Veterinary Medicine, Kafrelsheikh University, Kafrelsheikh, Egypt; 2 Department of Clinical Pathology, Faculty of Veterinary Medicine, Kafrelsheikh University, Kafrelsheikh, Egypt; 3 Department of Microbial Biotechnology, Genetic Engineering and Research Institute, University of Sadat City, Sadat City, Egypt; 4 Biology Department, College of Science, Princess Nourah Bint Abdulrahman University, Riyadh, Saudi Arabia; 5 Bioproducts Research Chair, Zoology Department, College of Science, King Saud University, Riyadh, Saudi Arabia; 6 Botany and Microbiology Department, Faculty of Science, Beni-Suef University, Beni-Suef, Egypt; 7 Department of Biology, College of Sciences and Arts-Khulais, University of Jeddah, Jeddah, Saudi Arabia

**Keywords:** Biochemistry, Biotechnology

## Abstract

Paracetamol is extensively consumed as an analgesic and antipyretic drug, but at a high dose level, it leads to deleterious side effects, such as hepatic and nephrotoxicity. This research aimed to estimate the prophylactic efficacy of *Chlorella vulgaris* and/or thiamine against paracetamol (P) induced hepatorenal and cardiac toxicity. Forty-eight female Wistar rats were randomly divided into eight equal groups (n = 6 rats). **Group 1**, normal control group. **Group 2**, Paracetamol group. **Groups 3**,** 4** and** 5** were treated with Silymarin drug, *Chlorella vulgaris* alga, *Chlorella vulgaris* alga supplemented with thiamine, respectively daily for 7 successive days, then all were administered Paracetamol (2gm/kg. bwt.). While, **Groups 6**, **7** and **8** were treated by Silymarin, *Chlorella vulgaris alga, Chlorella vulgaris* supplemented with thiamine, respectively daily for 7 successive days without paracetamol administration. Our results clarified that Paracetamol toxicity caused significant adverse effects on hematological, serum biochemical parameters, and oxidant -antioxidant status as well as histopathological picture of heart, liver, and kidney. However, in the Paracetamol intoxicated groups pretreatment either with *Chlorella vulgaris* alone or plus thiamine successfully improved the undesirable deleterious effects of paracetamol, and restored almost all variables to near their control levels. This study has finished to that oxidative stress participates in the pathogenesis of paracetamol-induced toxicity in rats and using *Chlorella vulgaris* alga either alone or plus thiamine alongside their health benefits can protect against oxidative harmful effects induced by paracetamol through their free radical scavenging and powerful antioxidant effects, and they can be used as propylactic agents against paracetamol-induced toxicity.

## Introduction

Acetaminophen (paracetamol, N-acetyl p-aminophenol; APAP) is a non-toxic and active analgesic/antipyretic at therapeutic levels. Moreover, paracetamol is metabolized at therapeutic doses, by phase II conjugating enzymes, mostly UDP-glucuronosyl transferase (UGT) and sulfotransferase (SULT), changing it to safe compounds which are secreted via the kidney. Just a very little portion is expelled in the urine. The residual paracetamol about five to nine percentage is biotransformed by the cytochrome P450 enzymes (CYPs), mostly CYP 2E1 into the highly reactive intermediate metabolite N-acetyl-p-benzoquinone imine (NAPQI)^1^. When the toxic dose of paracetamol is ingested, excessive NAPQ1 is produced and consequently causes serious GSH reduction as well as overproduction of reactive metabolites leading to covalent attachment of sulfhydryl groups in cellular proteins. This produces disrupts homeostasis and starts apoptosis or programmed cell death, leading to tissue necrosis and eventually to organ dysfunction which leads to liver oxidative stress^1,2^. Acute renal failure appears in nearly 1–2% of patients with acetaminophen overdose, in addition to hepatic necrosis^3,4^. Recently, the usage of natural substances for the prevention and treatment of liver disorders has increased^5^. Much attention has been pointed towards the application of natural antioxidants originated from plants for alleviating the oxidative damages produced by free radicals. Currently, numerous medicinal plants have shown such effectiveness^6^. Seeds of milk thistle (*Silybum marianum* L. Gaertn) have been used the extraction of a mixture of flavonolignans (Silymarin). Silymarin is a medicine used for the treatment of chronic and acute liver diseases^7^. The main actions of Silymarin are the scavenging of radical forms of oxygen and the stoppage of peroxynitrite creation^8^. Silymarin has been used as a protective drug against paracetamol-induced hepatotoxicity and nephrotoxicity due to its anti-inflammatory and antioxidant activities^9–11^.

*Chlorella vulgaris* is a single-cell green alga characterized by easy cultivation with high productivity and composed of superior contents of chlorophyll, lutein, protein, and many other necessary micro-nutrients^12,13^, *C. vulgaris* is documented as safe alga by the FDA^14^. It is considered as superfood including, 60% protein, 20 vitamins, 18 amino acids, and elements such as iron, potassium, calcium, phosphorous and magnesium^15^. Furthermore, there are many valuable antioxidants in microalgae, e.g., chlorophyll, carotenoids, astaxanthin, lutein, and phycobiliproteins^16,17^. *Chlorella* sp. supplementation revealed beneficial physiological effects such as antihypertensive^18^, antoxidative^19^, hypocholesterolemic^20^, and antitumor activities^21^, hypoglycemic and hypolipidemic effects^22,23^ in animal, and human studies. *Chorella* had hepatoprotective effect against carbon tetrachloride-induced liver damage in rats and mice^24,25^. Another alga as *Spirulina* showed potential a hepatoprotective and antioxidant activity against paracetamol-induced hepatic injury in rats^26^.

Thiamine is the active form of vitamin B1 that assists as a coenzyme in a number of the main metabolic pathways^27^. Zhou et al^28^ reported that, thiamine can reduce oxidative stress. Also, Asensi Fabado and Munne-Bosch^29^, stated that**,** the antioxidant action of thiamine can be indirect, by offering NADH and NADPH to the antioxidant network, or direct, by acting as an antioxidant. Thiamine Pyrophosphate proved to be as efficacious as standard therapy and may be beneficial in APAP-induced hepatotoxicity^30^.

However, the hepatorenal protective activity of *Chlorella vulgaris* is not extensively studied^31^. Therefore, the object of this study was to assess the protective effect or role of *Chlorella vulgaris* and/or thiamine against Paracetamol induced toxicity in rats. For this purpose, hematological, serum biochemical, tissues' lipid peroxidation, and antioxidant biomarkers and histopathological examinations were estimated in Paracetamol intoxicated Wistar rats pretreated either by *C. vulgaris* alga and /or thiamine.

## Results and discussion

### Body weight and weight gain changes

There was a significant (*p* ≤ 0.05) elevation in the final body weight and body weight gain in G5 and G8 followed by G4, and G7 compared to the normal control group (G1). While non-significant variations in the final body weight and body weight gain were seen in G2, G3 and G6 compared to the normal control group (G1) (Table 1).

**Table 1:** The changes in the body weight and body weight gain in the normal control and different treated rat groups.

Parameter group	Initial body wt	Final body wt	Body weight gain
G1	134.20 ± 2.22a	142.80 ± 2.63c	8.60 ± 0.07c
G2	133.80 ± 2.70a	143.40 ± 2.65c	9.60 ± 0.07c
G3	133.60 ± 2.24a	145.00 ± 2.23c	11.40 ± 0.07c
G4	133.40 ± 2.58a	147.20 ± 1.65b	13.80 ± 0.07b
G5	134.40 ± 2.33a	152.20 ± 3.13a	17.80 ± 0.07a
G6	134.40 ± 2.29a	145.20 ± 3.61c	10.80 ± 0.07c
G7	135.50 ± 2.54a	148.80 ± 1.68b	13.80 ± 0.07b
G8	134.80 ± 3.12a	153.20 ± 2.87a	18.40 ± 0.07a

G1 = control group, G2 = Paracetamol, G3 = Silymarin + Paracetamol, G4 = Chlorella vulgaris + Paracetamol, G5 = Chlorella vulgaris + Thiamine + Paracetamol, G6 = Silymarin, G7 = Chlorella vulgaris and G8 = Chlorella vulgaris + thiamine.

Data are presented as means ± SEM (n = 6). Values having different superscripts within same column are significantly different (*p* < 0.05).

### Absolute and relative organ weights

As demonstrated in (Table 2), there was a significant (*p* ≤ 0.05) increase in the absolute and relative weights of liver, kidney, and heart in paracetamol intoxicated group (G2) in comparison with control normal group (G1). Meanwhile, a significant (*p* ≤ 0.05) decrease in these organ weights was detected in G3, G4, and G5 compared with paracetamol intoxicated group (G2), the best reduction in these organ weights was seen in G3 and G5. On the other hand, groups G6, G7, G8 showed non-significant changes in kidney, liver, and heart weights in comparison with control normal group (G1).

**Table 2:** The changes in the absolute and relative weight of different organs of normal control and different treated rat groups.

Parameter group	Final body wt	Absolute wt			Relative wt		
		Liver	Kidney	Heart	Liver	Kidney	Heart
G1	142.80 ± 2.63	5.26 ± 0.28b	1.52 ± 0.03d	1.15 ± 0.05d	3.68 ± 0.14b	1.07 ± 0.01d	0.80 ± 0.02d
G2	143.40 ± 2.66	7.28 ± 0.28a	2.19 ± 0.07a	1.96 ± 0.05a	5.09 ± 0.23a	1.53 ± 0.03a	1.36 ± 0.03a
G3	145.00 ± 2.24	5.66 ± 0.25b	1.85 ± 0.07bc	1.45 ± 0.09 cd	3.89 ± 0.15b	1.27 ± 0.03b	1.00 ± 0.06 cd
G4	147.20 ± 1.66	6.05 ± 0.05b	1.94 ± 0.02ab	1.69 ± 0.10abc	4.11 ± 0.04b	1.32 ± 0.02b	1.14 ± 0.06abc
G5	152.20 ± 3.14	5.91 ± 0.02b	1.84 ± 0.05bc	1.47 ± 0.07 cd	3.89 ± 0.09b	1.21 ± 0.02bc	0.97 ± 0.04 cd
G6	145.20 ± 3.61	5.28 ± 0.32b	1.61 ± 0.07 cd	1.23 ± 0.09d	3.63 ± 0.13b	1.11 ± 0.02 cd	0.85 ± 0.05d
G7	148.80 ± 1.69	5.65 ± 0.11b	1.58 ± 0.04 cd	1.26 ± 0.05d	3.79 ± 0.09b	1.06 ± 0.02d	0.85 ± 0.03d
G8	153.20 ± 2.87	5.83 ± 0.05b	1.66 ± 0.09 cd	1.23 ± 0.04d	3.81 ± 0.04b	1.08 ± 0.04d	0.80 ± 0.03d

G1 = control group, G2 = Paracetamol, G3 = Silymarin + Paracetamol, G4 = Chlorella vulgaris + Paracetamol, G5 = Chlorella vulgaris + Thiamine + Paracetamol, G6 = Silymarin, G7 = Chlorella vulgaris and G8 = Chlorella vulgaris + thiamine.

Data are presented as means ± SEM (n = 6). Values having different superscripts within same column are significantly different (*p* < 0.05).

### Hematological parameters

The influences of paracetamol intoxication as well as the preventive effects of *C. vulgaris* and /or thiamine on hematological parameters of rats are shown in (Tables 3, 4). Paracetamol intoxication significantly (*p* ≤ 0.05) reduced RBCs count, Hb concentration, PCV%, platelets count, TLC, and neutrophils % with significant (*p* ≤ 0.05) rise in lymphocytes % in comparison with control normal group (G1). This picture was significantly (*p* ≤ 0.05) improved in the other treated groups compared with the paracetamol group (G2). The best improvement was detected in G3 and G5. Moreover, a significant increase in neutrophils % was observed in G8 compared with control (G1) and other treated groups.

**Table 3:** Erythrogram changes in the blood of normal control and different treated rat groups.

Parameter group	RBCs (106/ul)	HB (g/dl)	PCV (%)	Plateletes (103/ul)
G1	7.86 ± 0.49a	13.76 ± 0.49ab	41.66 ± 1.69ab	827.00 ± 35.75a
G2	5.01 ± 0.73c	10.86 ± 0.46c	32.94 ± 1.37c	421.60 ± 28.68c
G3	7.39 ± 0.43a	12.92 ± 0.28b	39.48 ± 0.79b	776.80 ± 56.17ab
G4	6.71 ± 0.21abc	12.12 ± 0.07bc	38.12 ± 0.18bc	773.40 ± 43.45ab
G5	6.92 ± 0.54ab	13.00 ± 0.25b	40.32 ± 1.01b	793.60 ± 49.64ab
G6	7.92 ± 0.54a	13.58 ± 0.52b	42.08 ± 1.42b	803.80 ± 29.06ab
G7	8.08 ± 0.12a	14.04 ± 0.19a	41.22 ± 1.03b	802.20 ± 32.92ab
G8	8.95 ± 0.30a	15.28 ± 0.37a	45.08 ± 0.82a	847.40 ± 35.25a

G1 = control group, G2 = Paracetamol, G3 = Silymarin + Paracetamol, G4 = Chlorella vulgaris + Paracetamol, G5 = Chlorella vulgaris + Thiamine + Paracetamol, G6 = Silymarin, G7 = Chlorella vulgaris and G8 = Chlorella vulgaris + thiamine. RBCs = Red blood cells, HB = Hemoglobin, PCV = Packed cell volume. Data are presented as means ± SEM (n = 6). Values having different superscripts within same column are significantly different (*p* < 0.05).

**Table 4:** Leukogram changes in the blood of normal control and different treated rat groups.

Parameter group	TLC (103/ul)	Neutrophils (%)	Lymphocytes (%)	Monocytes (%)
G1	8.00 ± 0.56ab	22.20 ± 1.82b	65.40 ± 1.74c	5.800 ± 0.58a
G2	5.12 ± 1.02c	19.40 ± 0.92c	73.20 ± 1.11a	5.800 ± 0.58a
G3	8.32 ± 0.23ab	23.20 ± 1.82b	68.80 ± 1.71bc	5.000 ± 0.70a
G4	8.44 ± 0.70ab	23.20 ± 1.71b	69.40 ± 0.24bc	5.600 ± 0.92a
G5	8.62 ± 0.49ab	24.00 ± 1.34b	66.20 ± 1.46c	6.000 ± 0.70a
G6	8.68 ± 0.78ab	23.40 ± 1.88b	66.40 ± 1.56c	6.200 ± 0.86a
G7	8.20 ± 0.48ab	23.20 ± 1.59b	66.60 ± 1.60c	6.000 ± 0.70a
G8	8.98 ± 0.31a	26.40 ± 0.81a	66.40 ± 1.53c	5.800 ± 0.58a

G1 = control group, G2 = Paracetamol, G3 = Silymarin + Paracetamol, G4 = Chlorella vulgaris + Paracetamol, G5 = Chlorella vulgaris + Thiamine + Paracetamol, G6 = Silymarin, G7 = Chlorella vulgaris and G8 = Chlorella vulgaris + thiamine. TLCs = Total leukocyte counts.

Data are presented as means ± SEM (n = 6). Values having different superscripts within same column are significantly different (*p* < 0.05).

### Serum biochemical parameters

**Figure 1:**
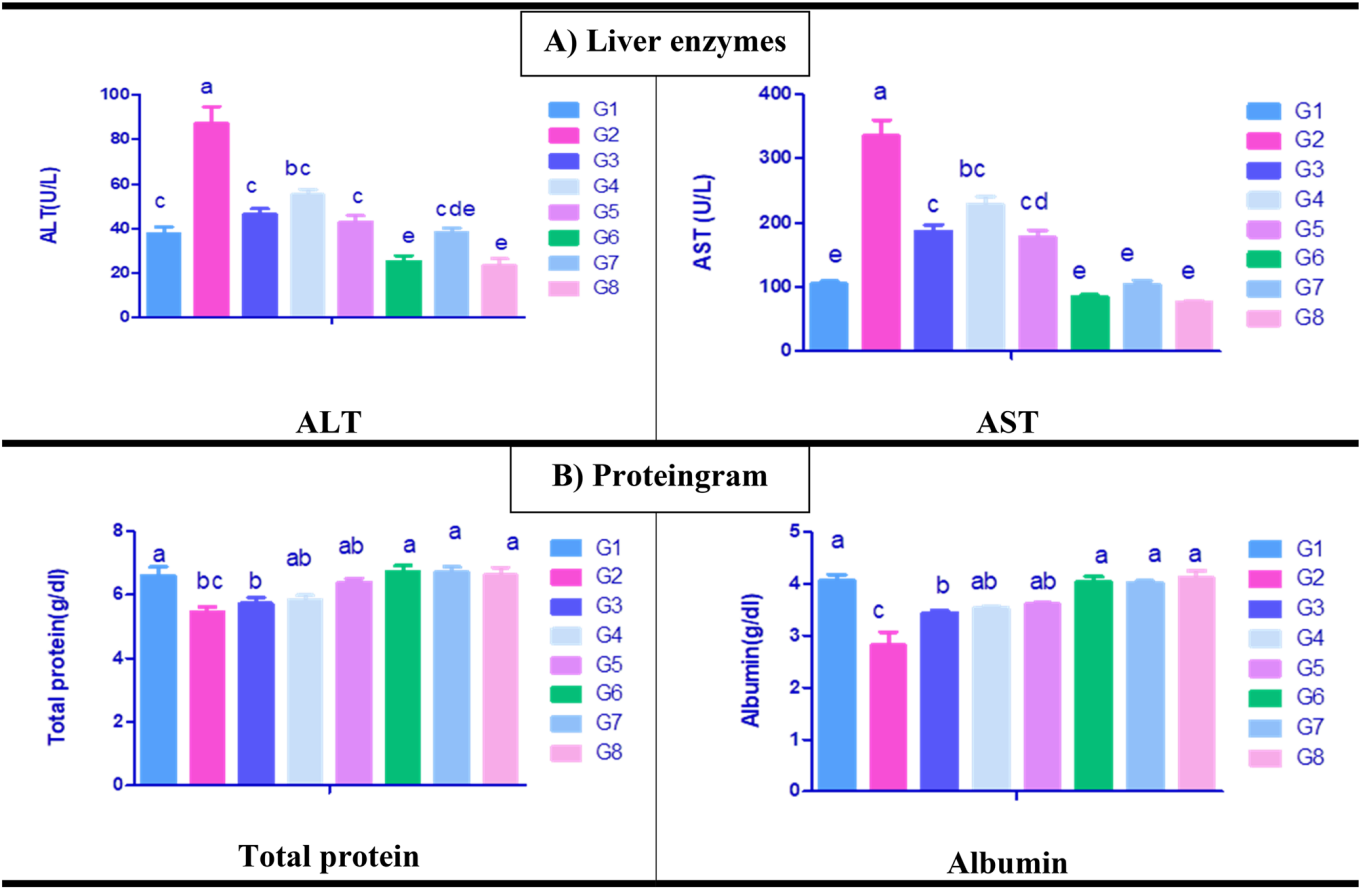
Serum biochemical parameters of liver enzymes and proteinogram of control and different treated rat groups. G1 = Control group, G2 = Paracetamol, G3 = Silymarin + Paracetamol, G4 = *Chlorella vulgaris* + Paracetamol, G5 = *Chlorella vulgaris* + Thiamine + Paracetamol, G6 = Silymarin, G7 = *Chlorella vulgaris,* G8 = *Chlorella vulgaris* + Thiamine. ALT = Alanine amino transferase, AST = Aspartate amino transferase. Data are presented as means ± SEM (n = 6). Different letter means significant difference effects in the same time period.

**Figure 2:**
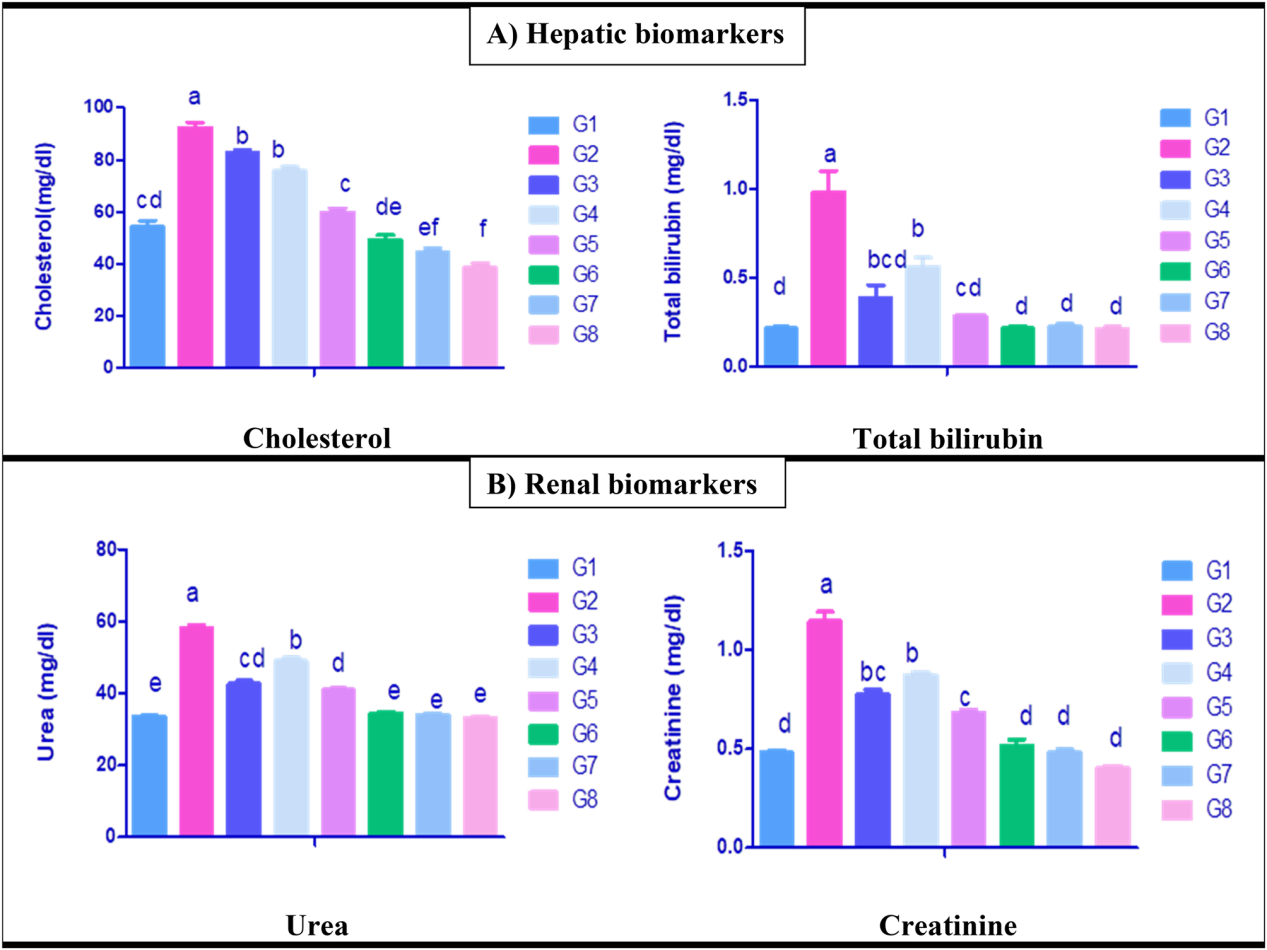
Serum biochemical parameters of hepatic and renal biomarkers of control and different treated rat groups. G1 = Control group, G2 = Paracetamol, G3 = Silymarin + Paracetamol, G4 = *Chlorella vulgaris *+ Paracetamol, G5 = *Chlorella vulgaris* + Thiamine + Paracetamol, G6 = Silymarin, G7 = *Chlorella vulgaris*, G8 = *Chlorella vulgaris* + Thiamine. Data are presented as means ± SEM (n = 6). Different letter means significant difference effects in the same time period.

The influences of paracetamol induced toxicity and the protective effects of *C. vulgaris* and /or thiamine on serum biochemical parameters are shown in (Figs. 1A,B, 2A,B). Paracetamol exposed rats group (G2) revealed significantly increased serum transaminases activities (Fig. 1A), cholesterol, bilirubin levels (Fig. 2A) as well as elevated urea, and creatinine levels (Fig. 2B) with significant decline in serum total protein and albumin concentrations (Fig. 1B) in comparison with normal control rats group (G1). Moreover, a significant alleviation in the same parameters was seen in G3, G4 and G5 compared with paracetamol exposed group (G2), the best improvement was observed in G3 and G5. However, a significant decline in ALT activity was shown in G6, G7 and G8 compared with normal control rat (G1). Meanwhile, a significant reduction in cholesterol was seen in G7 and G8 in comparison to normal control rat group (G1).

### Hepatic renal and cardiac antioxidant status and lipid peroxidation

**Figure 3:**
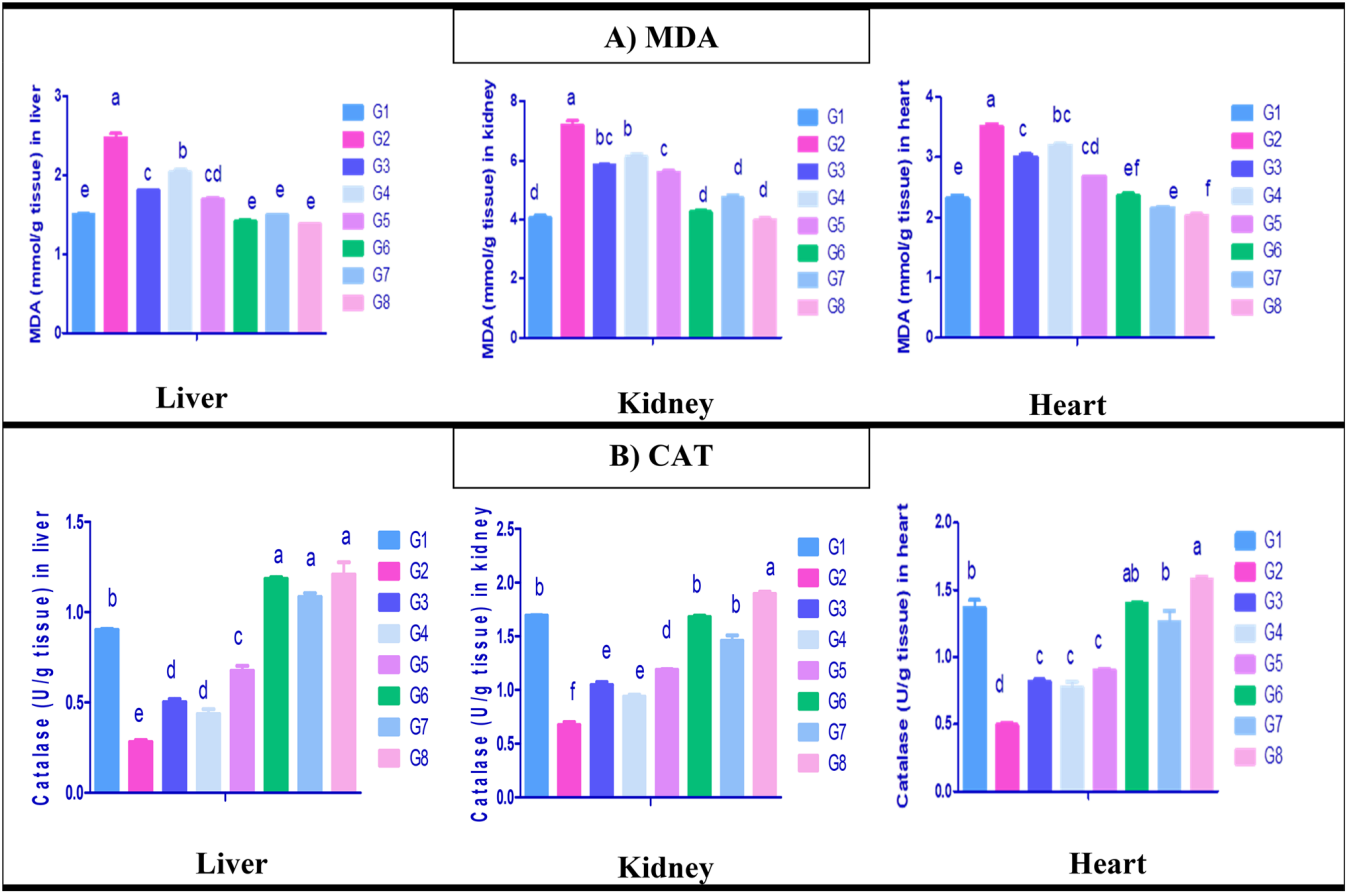
Oxidative stress and antioxidant status. (**A**) Malondialdehyde (MDA) (nmol/gm tissue), (**B**) Catalase activity (CAT) (μmol/mg) of liver, kidney and heart tissues of control and different treated rat groups. G1 = Control group, G2 = Paracetamol, G3 = Silymarin + Paracetamol, G4 = *Chlorella vulgaris* + Paracetamol, G5 = *Chlorella vulgaris* + Thiamine + Paracetamol, G6 = Silymarin, G7 = *Chlorella vulgaris*, G8 = *Chlorella vulgaris* + Thiamine. Data are presented as means ± SEM (n = 6). Different letter means significant difference effects in the same time period.

The influences of paracetamol induced toxicity and administration of *C. vulgaris* and /or thiamine on the lipid perioxidation and antioxidant enzymes of hepatic, renal, and cardiac tissues are shown in (Fig. 3A,B). MDA concentrations in hepatic, renal, and cardiac tissues were significantly elevated in paracetamol intoxicated group (G2) in comparison with the normal control group (G1) (Fig. 3A). Moreover, a significant decrease in hepatic, renal and cardiac MDA was observed in G3, G4, and G5 compared with paracetamol intoxicated group (G2). The superior reduction was observed in G3 and G5. On the other hand, paracetamol intoxication induced oxidative stress in liver, kidney and heart which resulted in the depletion of hepatic, renal and cardiac CAT activity (Fig. 3B). Furthermore, a significant elevation in catalase enzyme activity was detected in G3, G4 and G5 compared with paracetamol intoxicated group (G2), and the best was G5. Meanwhile, a significant increase in catalase activity was noticed in hepatic, renal, and cardiac tissues of rat groups administered *C. vulgaris* plus thiamine (G8) group compared to the normal control rat group (G1).

### Histopathological findings

**Figure 4:**
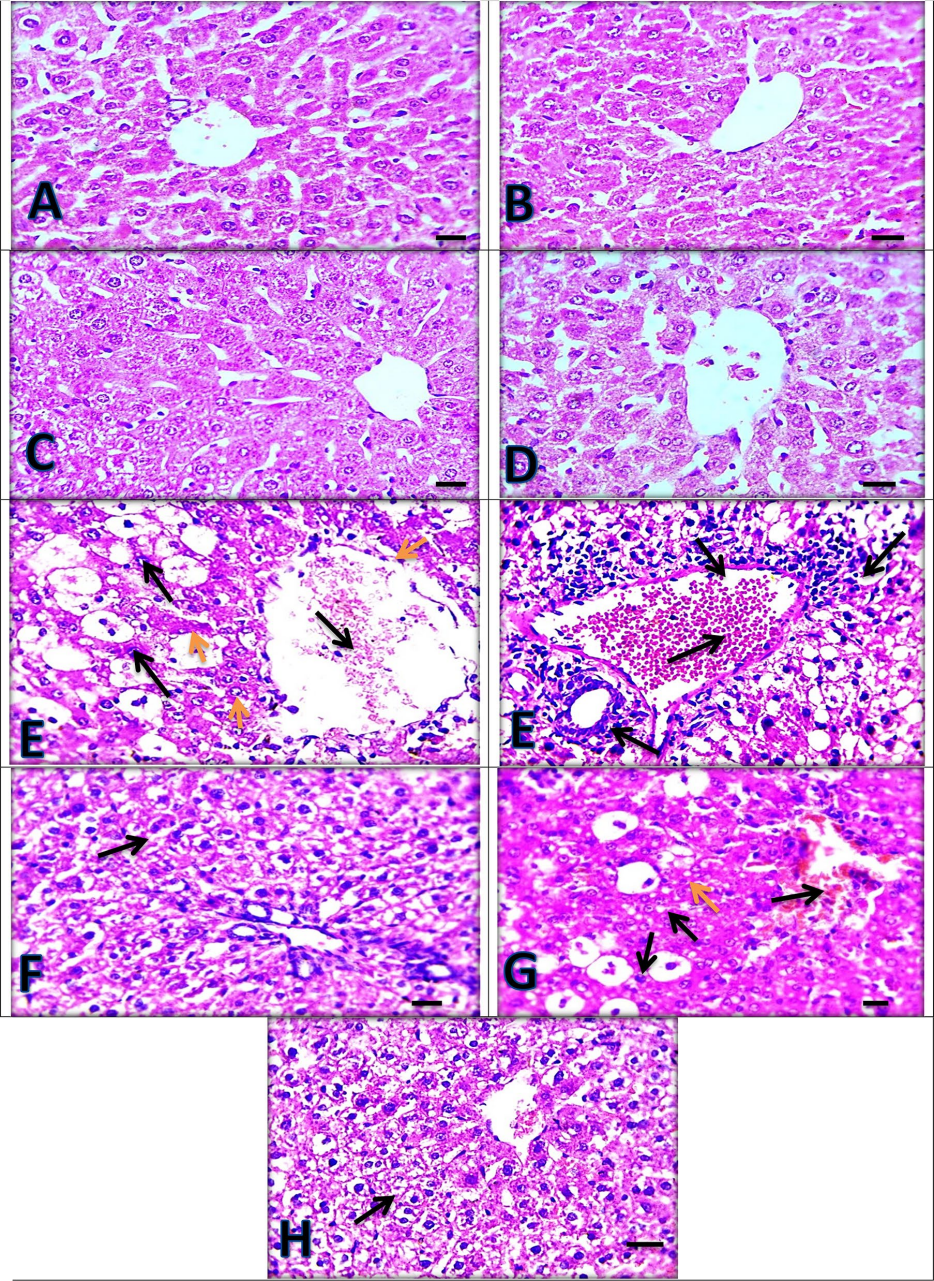
Liver sections showing normal appearance in (**A**) Control group, (**B**) Silymarin group, (**C**) *Chlorella vulgaris* group and (**D**) *Chlorella vulgaris* + thiamine group. (**E**) Paracetamol group showing severe congestion (black thin arrow) with marked vacuolar (yellow arrowheads) and ballooning degeneration in hepatocytes (black arrowheads) besides aggregation of lymphocytes in portal area (thick arrows). (**F**) Silymarin + Paracetamol group and (**H**)* Chlorella vulgaris* + Thiamine + Paracetamol group showing mild hydropic degeneration in hepatocytes (arrows). (**G**) *Chlorella vulgaris* + Paracetamol group showing moderate congestion (black thin arrow) vacuolar (yellow arrowheads) and ballooning degeneration (black arrowheads) in hepatocytes. (**H**) and (**E**) X: 400 bar 50.

Normal control rat group liver sections (Fig. 4A) showed normal hepatic architecture with no pathological changes. The same picture was seen in silymarin, *C. vulgaris* and *C. vulgaris* + thiamine treated groups, respectively (Fig. 4B–D) confirming the hepatoprotective effects of silymarin, *C. vulgaris* and thiamine when they were administered separately*.* Moreover, the paracetamol intoxicated group (Fig. 4E) revealed severe congestion, and most of the centrilobular hepatocytes showed marked vacuolar and ballooning degeneration, besides aggregation of lymphocytes in the portal area. The hepatic structure was improved and looked close normal with mild hydropic degeneration in hepatocytes in silymarin + paracetamol group and *C. vulgaris* + thiamine + paracetamol group (Fig. 4F,H). Moreover, paracetamol intoxicated rats given *C. vulgaris* showed moderate congestion, vacuolar and ballooning degeneration in hepatocytes (Fig. 4G).

**Figure 5:**
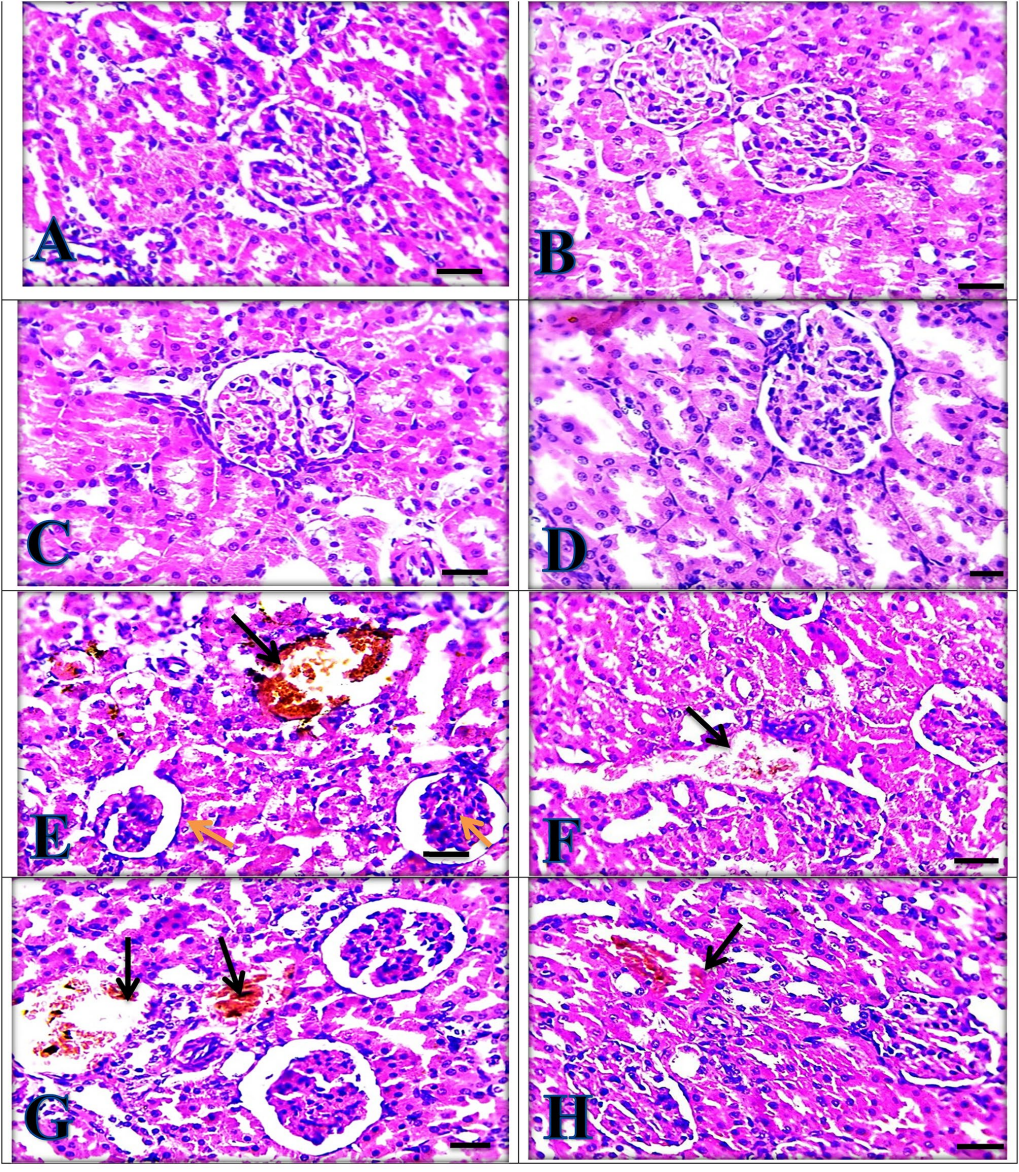
Kidney sections showing normal appearance in (**A**) control group, (**B**) Silymarin group, (**C**) *Chlorella vulgaris* group and (**D**) *Chlorella vulgaris* + Thiamine group. (**E**) Paracetamol group showing severe congestion (black arrow) and glomerular shrinkage (yellow arrows). (**F**) Silymarin + Paracetamol group and (**H**) *Chlorella vulgaris* + *Thiamine* + Paracetamol group showing mild congestion (black arrow). (**G**) *Chlorella vulgaris* + Paracetamol group showing moderate congestion (black arrow). (**H**) and (**E**) X: 400 bar 50.

Kidney sections showed normal appearance of the glomerulus and tubules of control group (Fig. 5A). The same picture was seen in silymarin, *C. vulgaris* and *C. vulgaris* + thiamine treated groups, respectively (Fig. 5B–D), confirming the nephroprotective effects of silymarin, *C. vulgaris,* and thiamine when given separately*.* Paracetamol intoxicated group (Fig. 5E) showed severe congestion, marked tubular dilation with loss of cellular boundary and epithelial degeneration , glomerular shrinkage, bleeding and partial endothelial rupture in capsule. Silymarin and *C. vulgaris* + Thiamine administrations to paracetamol intoxicated groups revealed mild congestion (Fig. 5F,H). While,* C. vulgaris* + paracetamol group was showed the moderate congestion beside the moderate tubular dilation as in (Fig. 5G).

**Figure 6:**
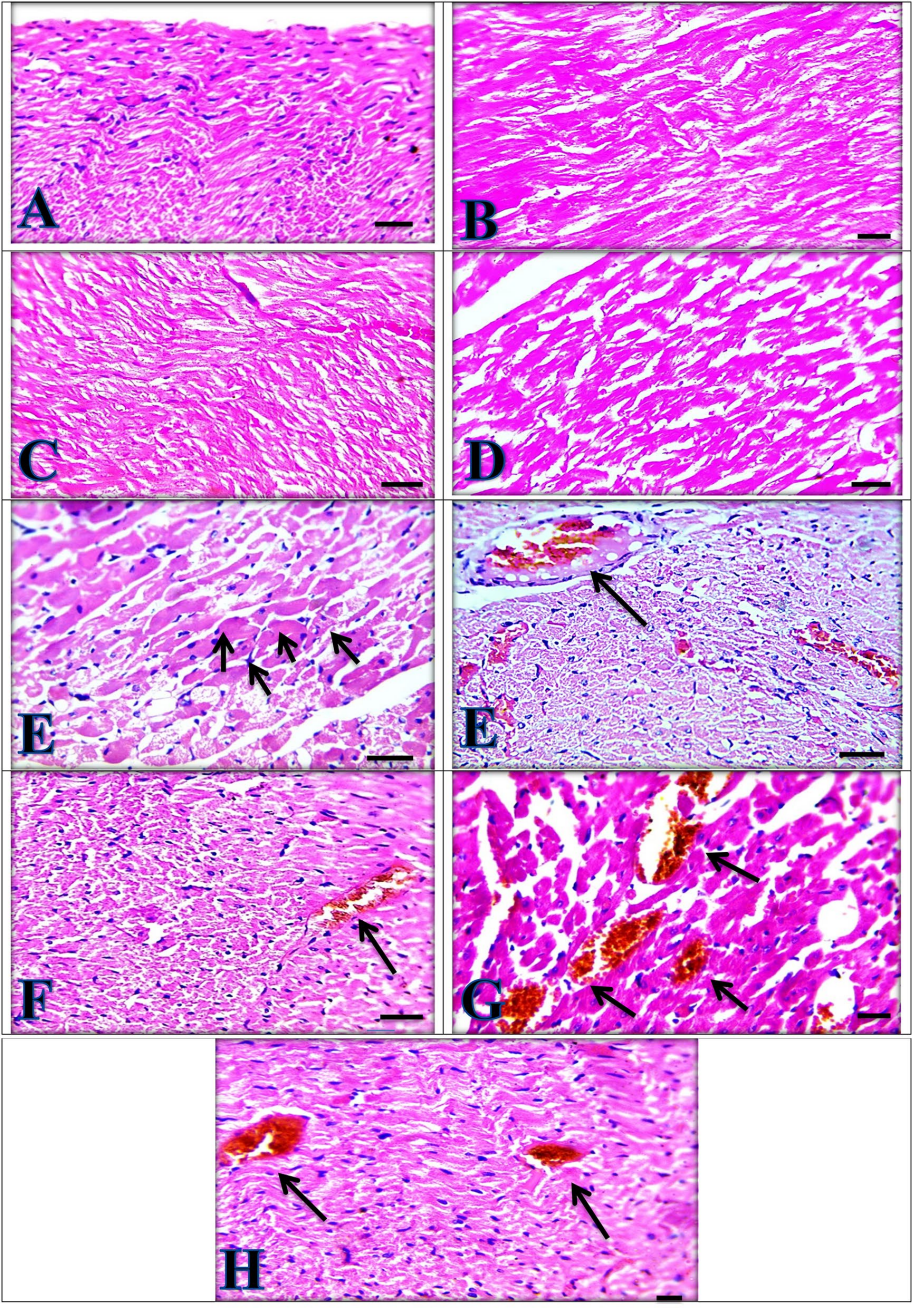
Heart sections showing normal appearance in (**A**) Control group, (**B**) Silymarin group, (**C**) *Chlorella vulgaris* group (**D**)* Chlorella vulgaris* + *Thiamine group*. (**E**) Paracetamol group showing severely congested cardiac blood vessels (arrows) besides degeneration and vacuolation in cardiomyocytes (arrowheads). (**F**) Silymarin + Paracetamol group and (**H**)* Chlorella vulgaris* + Thiamine + Paracetamol showing mildly congested cardiac blood vessels (arrows). (**G**)* Chlorella vulgaris* + Paracetamol group showing moderately congested cardiac blood vessels. (**H**) and (**E**) X: 400 bar 50.

Heart sections showed the normal appearance of cardiomyocytes of control rat group (Fig. 6A). The same findings were detected in silymarin, *C. vulgaris* and *C. vulgaris* + thiamine treated groups, respectively (Fig. 6B–D). On the other hand, the paracetamol intoxicated group showed degeneration and vacuolation in cardiomyocytes with severely congested cardiac blood vessels (Fig. 6E). This lesion was much improved by the administration of either Silymarin or *C. vulgaris* plus Thiamine to the paracetamol intoxicated groups which showed mild congestion, respectively (Fig. 6F,H). While moderate congestion was seen by* C. vulgaris* administration to paracetamol intoxicated group (Fig. 6G).

### FT-IR

FT-IR technique was used for evaluation the type of organic and inorganic complexes in *Chlorella vulgaris* and *C. vulgaris* supplemented with Thiamine. The FT-IR analyzes of *C. vulgaris* biomass without any addition (control) and *C. vulgaris* supplemented with thiamin represented different absorption peeks. The peeks with *C. vulgaris* control were 3404, 2970, 2925, 2856, 1655, 1549, 1408, 1384, 1054, 711 and 568 cm^−1^ which has shifted to 3449, 2959,2954, 2853, 2768, 1646, 1384, 1076, 875, 831, 600 and 564 cm^−1^. The infra-red spectrum displays a frequency ranges from 3500 to 3200 cm^−1^ indicating the O–H stretching vibration, existence of alcohols, phenols. The frequency ranges from, 3000–2850 cm^−1^peaks are representing in the C-H stretching vibration existence of alkenes. The wavenumber of some peaks of *C. vulgaris* biomass were decreased or increased after supplemented with thiamine such as wavenumber peak at 3404 increased to 3449, the peak 2970 decreased to 2959, peaks at 2925 increased to 2954, peak 2856 decreased to 2853, peak 1655 decreased to 1646, peak 1054 increased to 1076 respectively. There are some new peaks and also some peaks are disappeared as shown in Table 5, these results showed the difference in the alga compositions when supplemented with thiamine and hence its effect on oxidative stress induced by paracetamol.

**Table 5:** The FT-IR frequency range and the following functional groups are present in the C. vulgaris and with thiamine.

*C. vulgaris*	*C. vulgaris* with thiamine	Difference	Frequency ranges(cm^−1^)	Functional groups
3404	3449	54	3500–3200	O–H stretching vibration occurrence of alcohols, phenols
2970	2959	− 11	3000–2850	C–H stretching vibration occurrence of alkenes
2925	2954	29	3000–2850	C–H stretching vibration occurrence of alkenes
2856	2853	− 3	3000–2850	C–H stretching vibration occurrence of alkenes
–	2768	–	2925–2875	Aliphatic C–H Stretching vibration
1655	1646	− 9	1680–1640	–C=C– stretching vibration
1549	–	–	1550–1475	N–O asymmetric stretching vibration presence of nitro compounds
1408	–	–	1500–1400	C–C stretching vibration presence of aromatics
1384	1384	–	1390–1365	C–C stretching vibration presence of aromatics
1054	1076	22	1250–1020	C–N stretch stretching vibration
711	875–831	–	910–665	N–H wag stretching vibration
568	600–564	32–4	690–515	C–Br stretching vibration presence of alkyl halides

Oxidative stress is a phenomenon caused by an imbalance between production and accumulation of oxygen reactive species (ROS) in cells and tissues and the ability of a biological system to detoxify these reactive products. ROS can play, and in fact they do it, several physiological roles (i.e., cell signaling), and they are normally generated as by-products of oxygen metabolism; despite this, environmental stressors (i.e., UV, ionizing radiations, pollutants, and heavy metals) and xenobiotics (i.e., antiblastic drugs) contribute to greatly increase ROS production, therefore causing the imbalance that leads to cell and tissue damage (oxidative stress)^32^.

Oxidative stress plays a vital role in the pathogenesis of paracetamol induced liver damage^33^. This study demonstrated that paracetamol intoxication caused deleterious impacts on hemopoietic organs, which represented by lowered hematological parameters including, RBCs counts, Hb concentration, PCV%, TLC, Platelets count and neutrophil%. These findings are consistent with that of Desnoyers^34^;Taylor & Dhupa^35^ who demonstrated that the changes in the analyzed blood parameters might be due to the oxidative stress induced by paracetamol which has a damaging effect on immune and hemopoietic organs and erythrocytes. Paracetamol inhibits hemopoesis together with hematotoxicity, primarily methemoglobinemia and hemolytic anemia. This may be attributed to the destruction of RBCs by increased lipid peroxidation in cell membranes^36^. Moreover, uremia has a bad effect on blood platelets^37^. On the same line, Adedapo et al^38^, Daniel and Clement^39^, Biu et al^40^ reported that, xenobiotics intoxication exhibited potential inhibition of erythropoietin release from damaged kidneys and susceptibility of this highly proliferative tissue for toxicity. The current research declared that paracetamol stimulated hepatic renal and cardiac damage which was represented by alterations in the serum biochemical parameters. These alterations are implicated in a series of events leading to paracetamol mediated hepatic, renal and cardiac toxicities. Such toxicities are the consequences of the oxidative injuries induced by excessive generation of ROS and the impairment of the antioxidant enzyme activities. These results are in line with the previuos researches carried out by Nikravesh et al^41^, Zhao et al^42^ who reported that**,** lipid peroxidation and oxidative stress are the early events related to radicals generation during the hepatic metabolism of acetaminophen. Moreover, Du et al^2^ documented that the intracellular mechanisms of paracetamol-induced hepatocytic injury is by mitochondrial dysfunction and excessive ROS production causing severe oxidative stress. Paracetamol can stimulate liver injury by oxidative stress and inflammation^42,43^. Gini and Muraleedhara^44^; Kanchana and Sadiq^45^ concluded that overdose of paracetamol induces toxicity to the hepatocytes**.** Our results are also in harmony with Sabiu et al^46^ who indicated that cellular leakage and loss of functional integrity of the liver cell membrane duo to paracetamol intoxication revealed a significant increase in the serum enzyme activities of ALTand AST with elevation of bilirubin and cholesterol levels. Moreover, the significant elevation in cholesterol level recorded after paracetamol administration may be due to the imbalance between the normal rates of lipid synthesis, utilization and secretion^47,48^ or may be duo to inhibition of bile acid synthesis as recorded by previous studies^49–51^.

The reduced serum total protein and albumin concentrations following paracetamol overdose exposure in this study resulting from disturbance of protein synthesis as a consequence of altered hepatic function as a result of inflammation^52^ or due to nephrotoxicity which leads to leakage of albumin in urine with decreasing of serum albumin and total protein concentrations^53^.

Our study clearly demonstrates that acute acetaminophen toxicity enhanced renal MDA level, depleted the renal CAT antioxidant activity leading to elevated serum urea and creatinine levels, reduced total protein and deteriorated the renal architecture as confirmed by our histopathological observations. The end product of lipid peroxidation is MDA, which is recognized as the second messenger of free radicals. The high concentration of MDA in renal tissue denotes to renal toxicity^54^. Inconsistent with our results, Srinivasan et al^33^ who reported that, increased ROS level and decreased enzymatic antioxidants considered as a mechanism by which several chemicals can induce nephrotoxicity leading to disturbance of cell membrane integrity. Paracetamol nephrotoxicity occurs as a result of its highly reactive metabolite- NAPQI- which acrylates proteins in the proximal tubule, initiating renal tubular cells death^55^. In accordance with our results, Mandal et al^54^, Das et al^56^ who concluded that, acetaminophen overdose is often associated with elevation of urea and creatinine concentrations which are indicators of drug-induced nephrotoxicity in animals**.** In line with our observation Cohen et al^57^ who demonstrated that acetaminophen overdose decreased antioxidant enzymes in kidney tissues and enhanced lipid peroxidation. Similary, Jones and Vale^58^ reported that paracetamol overdose induced hepatic and renal deleterious necrosis in humans and experimental animals.

Several herbal and plant extracts derived compounds served as alternative therapeutic agents to counteract the side effects of various drugs^59,60^.

In the current study silymarin succeeded to overcome the deleterious impacts of paracetamol intoxication on rat hematological, biochemical parameters and histopathological changes, reduced hepatic, renal and cardiac oxidative damage and enhanced hepatic, renal and cardiac antioxidants. In consistent with our results, Papackova et al^8^ who pointed that the main actions of silymarin are scavenging of radical forms of oxygen and inhibition of peroxynitrite formation. Furthermore, Freitag et al^10^ stated that, the prophylactic activity of silymarin against paracetamol-induced hepatotoxicity is generally attributed to its antioxidant and anti-inflammatory properties. Several studies about the standard drug silymarin found that silymarin offered protection against chemical hepatotoxins such as CCl4, ethanol, and paracetamol^61^. Moreover, Cacciapuoti et al^62^ mentioned that silymarin is an effective remedy for decreasing hepatic steatosis in patients with non-alcoholic fatty liver disease. Silymarin was approved for the treatment of the hepatotoxic doses of paracetamol. Therefore, in this research we used silymarin as a standard control drug.

Regarding to the effect of *C. vulgaris* algae on body weight, our results showed a significant increase in final body weight and body weight gains in response to *C. vulgaris* algae administration in comparison to the control and other treated groups. *C. vulgaris* is a rich source for chlorophyll pigment and vital amino acids; in addition to considerable quantities of calcium, phosphorus, iodine, manganese, iron and vitamins such as A, B1, B2, B3, B6, B12, C 67 and E^63^. In agreement with our results, Xu et al^64^ who stated that *C. vulgaris* can be a useful choice as an additive for fish diets, they claimed that *C. vulgaris* could improve digestive the enzymes and enhance growth performance and immunity due to its high concentrations of the crude protein, polysaccharides, lipid, minerals and other bioactive components involved in many physiological activities**.** On the same line, Kang et al^65^ concluded that *Chlorella* additions to the diets of broiler chicks improved body weight.

Concerning to the effects of *C. vulgaris* against paracetamol intoxication, the current results demonstrated that rats administered *C. vulgaris* at the chosen dose either alone or with thiamine succeeded to minimize the deleterious effects of paracetamol on rats’ hematological, biochemical, antioxidant status and histopathological findings, suggested that *C. vulgaris* exhibits excellent hepato protective properties and has some role in maintaining the structural integrity of the hepatocellular membrane, thus preventing the enzymes leakage into the blood circulation, together with repairing of the hepatic tissue damage induced by paracetamol. This impact is in consistent with Ahmed and Khater^66^, Pawlikowska-Pawlega et al^67^ who stated that serum levels of transaminases returned to normal with the healing of hepatic parenchyma and the regeneration of hepatocytes**.** Moreover, Rodriguez-Garcia and Guil-Guerrero^68^ reported that *Chlorella vulgaris* exhibited antioxidative and hepatoprotective effects. Furthermore**,** Cheng et al^69^ recorded that the possible mechanism for *C. vulgaris* protection may be attributed to its immunomodulatory potential, that may stimulate the lymphocytes propagation and phagocytic activities of macrophages, promote the expressions of cytokines, improve the NK cells cytotoxicity, and ameliorate the histological changes of the spleen.

Furthermore, *C. vulgaris* succeeded to restore the levels of urea, and creatinine close to normal values thus preventing the kidney from damage. These restorative effects of *C. vulgaris* over the serum clinical chemistry correlate with previous studies used *C. vulgaris* for treating oxidative stress^31^. On the same line several studies declared that *Chlorella vulgaris* administration provided protection against membrane fragility with anti-inflammatory, antihypertensive, and antioxidative activities^18,19^. As *C. vulgaris* microalgae, contains many valuable antioxidants as chlorophyll, carotenoids, astaxanthin, lutein and phycobili-proteins^17^, with the highest amount of chlorophyll than any known plant. Moreover, *C. vulgaris* prevented the lipid peroxidation in hepatic, renal and cardiac tissues. In addition to, its ability to abolish the toxic effect of paracetamol on the examined tissues through increasing the activities of antioxidant enzymes. The protective effects of *C. vulgaris* and its antioxidant activity are attributed to their content of phenolic compounds^70^ as there is a close positive relationship or correlation between the quantity of these compounds in *C. vulgaris* extract and their antioxidant activities due to their redox properties that play a vital role in capturing and scavenging free radicals, oxygen suppression and peroxide decomposition^71–73^. Furthermore*, C. vulgaris* extract significantly decreased the degree of lipid peroxidation and TBARS level in leukocytes in comparison to *Ganoderma lucidum* extract in vitro location^74^**.** The same results were detected when *C. vulgaris* is supplemented alone or with thiamine. In agreement with our observation Zhou et al^28^ who reported that thiamine can reduce oxidative stress. Furthermore, Asensi Fabado and Munne-Bosch^29^ stated that**,** the antioxidant activities of thiamine can be indirect, by providing NADH and NADPH to the antioxidant network, or direct, by acting as an antioxidant.

The prophylactic effects of *C. vulgaris* against oxidative stress induced by paracetamol intoxication in our study could be due to inhibition of lipid peroxidation and scavenging of free radicals as its administration was responsible for the increased resistance against oxidative stress induced by paracetamol which consequently plays a fundamental role in the pathogenesis of paracetamol induced liver damage^33,52^. The elevated levels of MDA demonstrated in the present study are in accordance with those of other investigators who reported the association between paracetamol toxicity and MDA elevation^75^. Moreover, *C. vulgaris* and or thiamine prevented the lipid peroxidation in hepatic, renal and cardiac tissues and improved the activities of antioxidant enzymes in rats tissues, such effects could be the mechanisms of their hepatorenal protection. This is in agreement with the report of Sabiu et al^76^ who stated that acetaminophen mediated hepatic oxidative insults in rats had induced significant decrease in the activities of antioxidant enzymes**.**

Compared with the standard drug silymarin, no significant differences were detected in the protection induced by silymarin treatment and *C. vulgaris* and /or thiamine treatment, suggesting that *C. vulgaris* either alone or with thiamine succeeded to prevent disruption of organs function by protecting the lipids from peroxidation by ROS under paracetamol toxicity and enhancing antioxidant enzymes activity.

## Material and methods

### Chemicals

Paracetamol tablets (each tablet contains 500 mg) was obtained from El-Nasr Pharmaceutical Chemicals Co., Egypt. Paracetamol was suspended in pathogen-free normal distilled water prior usage. Silymarin capsules (Legalon 140) each capsule contains 140 mg was purchased from Ced Pharmaceutical Co, Giza, Egypt.

The diagnostic kits used for assaying hepatic and kidney performance tests, the levels of lipid peroxidation and antioxidants were obtained from Bio-Diagnostic Co., Giza, Egypt. All other chemicals used throughout the experiments were of high analytical grade. Thiamine powder was obtained from El-Nasr Pharmaceutical Chemicals Co, Egypt.

### *Chlorella vulgaris alga* (CV)

*Chlorella vulgaris alga* was obtained from «Microbial Biotechnology Lap, Genetic Engineering and Biotechnology Research Institute (GEBRI), University of Sadat City, Sadat City, Egypt^»^. BG11 nutritive culture was used as a medium for enrichment and growth of the tested alga.

### *Chlorella vulgaris alga* supplemented with *Thiamine*

Two hundred ml of the BG11 nutritive culture medium were prepared and supplemented with 0.08 mg/L vitamin B1 (thiamine)***,*** after sterilization* C. vulgaris* was inoculated. The culture was incubated at natural day light at temperature 30 ± 2 °C and shaken gently twice a day to avoid clumping and enhance the growth. After 15 days of incubations, the culture was centrifuged, washed by distilled water and dried through the hot air oven at 60 °C until the constant weight was obtained.

The required daily dose from *Chlorella* powder and *Chlorella* supplemented with thiamine administered to the animals in this study were dissolved in sterilized distilled water to be in suspension format using Ultrasonic homogenizer sonication (Biologics Inc. USA manufacturer and leading innovator)^77^.

### FT-IR analysis

FTIR spectroscopy is a technique required to define cell contents of microalgae^78^. FTIR spectra illustrates the macromolecular composition of the algal biomass depending on the infrared absorption of functional groups. So, FTIR spectroscopy permits the revealing of changes in the relative abundance of organic compounds such as carbohydrate, lipid and protein. This technique was used in many studies to define the changes in the macromolecular composition of microalgae caused by nutrient stress^79–82^. The change of functional groups present in dry algal biomass control and that supplemented with Thiamine has been described by Fourier transform infrared (FTIR) spectroscopy according to methods of Jebsen^83^.

### Animals and experimental design

This study was approved by the Research Ethical Committee of the Genetic Engineering and Research Institute, Sadat City University, Egypt. Forty eight female albino rats of Wistar strain (130–150 g) were obtained from the Animal House of the Genetic Engineering and Research Institute, University of Sadat City, Egypt and housed in well- ventilated plastic cages. The diet and water were provided ad-libitum. All rats were housed under standard husbandry conditions (25 ± 2 °C temp, 60 ± 5% relative humidity and 12 h photoperiod). Rats were kept untreated for two weeks for acclimatization prior treatment and were weighed at the starting of research (initial weight). All animal handling procedures, sample collection and disposal were according to the regulation of Institutional Animal Care and Use Committee (IACUC), Genetic Engineering and Research Institute University of Sadat City, Egypt, under approval number (gebriUSC-009-1-19).

Forty eight female albino rats of Wistar strain were randomly divided into eight equal groups (n = 6 rats each) as the following:

**Group 1,** Normal control group, it was administered distilled water only per os (0.5 ml/rat) daily for 7 successive days.

**Group 2,** Paracetamol group, it was treated as normal control group for 7 successive days, then given Paracetamol per os once (2gm/kg.bwt.). according to Sharoud^26^.

**Group 3,** Silymarin** + **Paracetamol group**,** it was treated with Silymarin drug (100 mg/kg. b.wt.) according to Bektur et al^9^ per os daily for 7 successive days, then administered Paracetamol per os once (2gm/kg.bwt.).

**Group 4,** *Chlorella vulgaris* alga + Paracetamol group, it was treated with *Chlorella vulgaris* alga (500 mg/kg. b.wt) according to Hsin-yi et al^24^; Sharoud et al^26^ per os daily for 7 successive days, then administered Paracetamol per os once (2gm/kg.bwt.).

**Group 5,** *Chlorella vulgaris* alga + thiamine + Paracetamol group, it was treated with *Chlorella vulgaris* alga plus thiamine (500 mg/kg. b.wt), respectively per os daily for 7 successive days, then administered Paracetamol per os once (2gm/kg.bwt.).

**Groups 6,** Silymarin group, it was treated with Silymarin (100 mg/kg. b.wt) per os daily for 7 successive days without paracetamol administration.

**Group 7,** *Chlorella vulgaris* alga group, it was treated with *Chlorella vulgaris* alga (500 mg/kg. b.wt.) per os daily for 7 successive days without paracetamol administration.

**Group 8,** *Chlorella vulgaris* alga + thiamine group, it was treated with *Chlorella vulgaris* alga plus thiamine (500 mg/kg.b.wt.) per os daily for 7 successive days without paracetamol administration.

Rats of all the experimental groups were anaesthetized and euthanized after 24 h of the last treatment for samples collection.

### Sampling

At the end of the experiment (24 h after paracetmol administration), rats in all groups were fasted overnight and weighed to calculate the final body weights and weight gain. Then, blood samples were obtained from each rat via retro orbital bleeding under light ether anaesthesia (Sigma Chem. Co., St Louis, Mo. U.S.A). Two blood samples were taken from each rat. One sample was put into a tube containing heparin as anticoagulant for hematological assessment. The other sample was put in a tube without heparin and allowed to coagulate, then centrifuged at 3000 for 15 min. The clear sera were collected and kept at − 20 °C for subsequent biochemical analysis. After blood samples collection, rats were euthanized by cervical dislocation for tissue samples collection. Liver, kidney and heart from each rat were carefully excised, weighed and immediately cleaned with normal saline solution (0.9% NaCl). Each tissue sample was divided into two parts. A part was kept at − 80 °C for Malondialdehyde (MDA) and catalase (CAT) activity estimations. The other part was fixed in 10% neutral buffer formalin solution for further histopathological examinations.

### Absolute and relative body and organ weights

Just before killings the rats at 8th day of experiment, final body weight of all rats in all experimental groups was calculated. The body gain was calculated from the difference between the body weight at the beginning and at the end of experiment. Upon being sacrificed or killed, the liver, kidney and heart were aseptically removed, weighed and their relative organ weights (ROW) were determined according to the equation of Aniagu et al^84^.

$${\text{ROW}} = \left[ {{\text{Absolute}}\;{\text{organ}}\;{\text{weight}}\left( {\text{g}} \right)/{\text{body}}\;{\text{weight}}\;{\text{of}}\;{\text{rat}}\;{\text{on}}\;{\text{sacrifice}}\;{\text{day}}\left( {\text{g}} \right)} \right] \times 100.$$

### Hematological analysis

The whole blood samples were utilized directly after collection for estimation of hematological parameters including the red blood cells (RBCs), hemoglobin (Hb) concentration and hematocrit value (PCV%), total leucocytes count (TLC), differential leukocyte counts and platelets (Plt) counts, by using automated blood cells counter with an Auto Hematology Analyzer (Sysmex F-800, Japan)^85^.

### Biochemical assays

The biochemical parameters of liver and renal injury biomarkers were estimated in the collected serum samples according to the manufacturer protocol. Serum enzymatic activities of aspartate amino transferase (AST) and alanine amino transferase (ALT) were assessed according to Reitman and Frankel^86^. Albumin (Alb) and total proteins (TP) according to Henry et al^87^. Serum cholesterol was measured according to Richmond^88^. Total bilirubin was analyzed according to Tietz^89^. Renal products; creatinine was estimated according to Larsen^90^, and urea according to Coulombe and Favreau^91^.

### Evaluation of oxidative stress and antioxidant biomarkers

Immediately after blood collection, the animals were euthanzied by cervical dislocation, then the liver, kidney and heart from each rat were immediately dissected out and weighed. A part from each organ was homogenized using glass homogenizer with ice cooled saline to prepare 25% W/V homogenate. This homogenate was centrifuged at 1700 rpm for 10 min; the supernatant was stored at – 80 °C until analysis. This supernatant was used for the colorimetrical estimations of hepatic, renal and cardiac malondialdehyde (MDA), the main end product of lipid peroxidation, according to the protocol of Esterbauer et al^92^*,* and catalase activity according to the method of Sinha^93^.

### Histopathological results

The other parts of liver, kidney and heart of the scarified rats were fixed in 10% buffered formalin. Then, dehydration, clearance and processing in paraffin were carried out. Tissue sectioning and staining with H&E were performed according to Bancroft et al^94^.

### Statistical analysis

All data were expressed as means ± S.E. and statistically analyzed by one-way ANOVA and Tukey’s post-hoc test multiple comparisons using Graphpad prism Version 5 software (Graph Pad Software Inc., USA).. Statistical significance was acceptable to a level of *p* ≤ 0.05.

## Conclusions

Oxidative stress plays essential role in paracetamol induced hepatorenal and cardiac toxicity. *C. vulgaris* is a potent antioxidant agent that was indicated to protect intoxicated rats against oxidative stress induced by paracetamol. This study, revealed that paracetamol exposure resulted in varying degrees of lipid peroxidation, depletion of the antioxidant enzymes activity and changes of hematological, biochemical parameters and histopathological architectures of the examined tissues. *C. vulgaris* and /or thiamine pre-exposure offered near complete protection in terms of blood and tissues changes, antioxidant enzymes activity and oxidative stress. Therefore, this study suggested that *C. vulgaris* is a promizing protective agent against paracetamol induced toxicity as ROS scavenger and a potential source of natural antioxidants.

## Data Availability

The research data used to support the findings of this study are included within the article (tables, figures).
